# The Role of Patient Education in Low Anterior Resection Syndrome: A Systematic Review

**DOI:** 10.1007/s13187-025-02593-3

**Published:** 2025-02-27

**Authors:** P. Tang, R. Tovel, H. Ong, David Proud, Adele Burgess, Eleanor Watson, Wui Yeat Chen, David Lam, Helen Mohan

**Affiliations:** 1 Department of Colorectal Surgery, Austin Health, 145 Studley Road, Heidelberg, Melbourne, VIC 3084 Australia; 2 University of Melbourne, Melbourne, Australia; 3 Peter MacCallum Cancer Centre, Melbourne, Australia

**Keywords:** Low anterior resection syndrome, Rectal cancer, Education

## Abstract

Low anterior resection syndrome (LARS) is a significant complication after sphincter-preserving rectal cancer surgery and negatively impacts a patient’s quality of life. Treatment of LARS is multimodal, and many treatments require engagement from the patient. Understanding the complex array of treatment options and the potential additive effects of different therapies can be difficult. This systematic review aims to assess the role of patient education in treatment of LARS in colorectal cancer patients. A systematic literature search was conducted on PubMed, EMBASE and Ovid Medline according to the PRISMA guidelines for articles published from 1946 to October 2023 and registered with PROSPERO: 42,023,434,601. Studies that reported on clinical outcomes of patients with LARS that underwent any educational intervention were included for this review. Results from five publications including 368 patients with LARS were reviewed for analysis. Three interventions were delivered to symptomatic patients postoperatively and two to all patients who underwent low anterior resection preoperatively. Five trial protocols were also included for analysis, including three preoperative and two postoperative interventions Education interventions were often delivered alongside co-interventions such as medications and pelvic floor physiotherapy. Four studies reported improvement of mean LARS scores and quality of life measures related to the education intervention and associated co-interventions. Patient education has a positive impact towards improving functional outcomes in patients with LARS following resection for rectal cancer. In practice, it is often used in conjunction with other interventions. Determining optimal content and delivery methods is a target for future research.

**Supplementary Information:**

The online version contains supplementary material available at 10.1007/s13187-025-02593-3.

## Introduction

With advances in surgical techniques and neoadjuvant therapy, the survival rate of sphincter-preserving procedures for rectal cancer has significantly improved. However, the increase in sphincter-preserving rectal surgery has given rise to an increased incidence of postoperative bowel dysfunction in survivors of rectal cancer [1].

Low anterior resection syndrome (LARS) refers to the constellation of symptoms that can occur after sphincter-preserving rectal cancer surgery, including faecal incontinence, urgency and frequency. As the diagnosis of LARS is a clinical one, there exist multiple measures of its severity. Many of these measures are patient reported, such as the LARS score and Wexner score, and some include objective measures such as anal physiology measurements. LARS can be graded using the LARS score system—major LARS (score 30–42), minor LARS (score 21–29) and no LARS (score 0–20). Over 44% of patients who have undergone sphincter-preserving rectal surgery will experience major LARS [2]. Higher mean LARS scores have been shown to have a negative impact of quality of life, especially on physical and social well-being of patients [1–5].

Currently, there is no established standardised treatment strategy for LARS given the highly variable nature of the condition. Treatment is mostly empirical and symptom-based, which can involve lifestyle and pharmacological measures such as dietary modifications and anti-diarrhoeal medications for minor LARS, and interventions such as colonic irrigation, pelvic floor rehabilitation and sacral nerve stimulation for major LARS [6, 7].

There is no doubt that each modality has a role to play in the treatment of LARS, seemingly in an additive fashion. When used to its fullest, this combination of interventions can lead to significant symptom improvements, with each modality contributing in increments [8].

Current information available to patients on LARS is lacking [9]. Patients with LARS that have been interviewed in previous studies have also expressed a desire for more education and counselling about the syndrome before and after their surgery [10, 11].

Previous studies assessing the impact of patient education for colorectal cancer patients with stomas have shown a positive effect on patients’ psychosocial needs and quality of life [12, 13]. This study aims to assess whether patient education can improve the quality of life of colorectal cancer patients with LARS.

## Methods

### Search Strategy

This systematic review was prepared according to the Preferred Reporting Items for Systematic Reviews and Meta-analyses (PRISMA) guidelines and registered with PROSPERO: 42,023,434,601. An electronic search of the literature was conducted on PubMed, MEDLINE and EMBASE for articles published from 1946 to October 2023. This search strategy is detailed in the Appendix. Hand searching of relevant literature was reviewed to identify additional studies.

### Inclusion and Exclusion Criteria

**Fig. 1:**
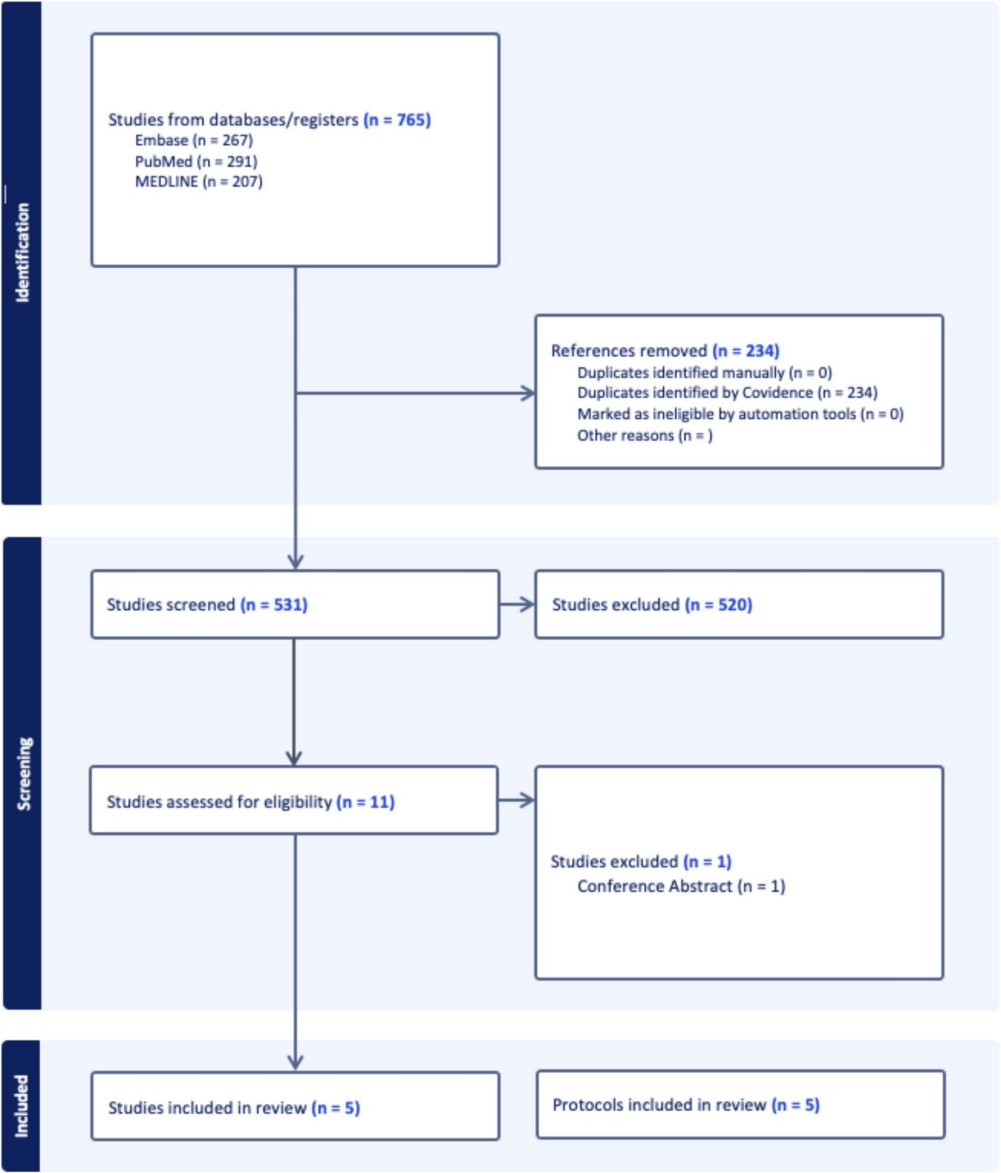
PRISMA diagram

All titles and abstracts retrieved from the search strategy were screened by two independent reviewers, and conflicts were resolved by a third reviewer. Both comparative and non-comparative studies that reported on clinical outcomes of patients with LARS that underwent any form of educational intervention were included for this review, e.g. if an intervention such as pelvic floor rehabilitation specified an education component in the study. Case reports and conference abstracts were excluded. This process is summarised in the PRISMA diagram in Fig. 1.

### Determining Study Quality

For non-RCTs, the risk of bias in non-randomised studies—of interventions (ROBINS-I) tool was used, which assesses risk of bias at the pre-intervention, at intervention and at post-intervention stages. For RCTs, the revised Cochrane risk-of-bias tool for randomised trials was used.

### Outcomes of Interest

The primary outcome of interest was the incidence of major LARS, as defined by the LARS score. Secondary outcomes include quality of life scores and whether further intervention was required.

## Results

### Study and Patient Characteristics

Five studies [14–18] have been included in this review, with 368 patients in total. Two studies were RCTs [14, 18] and the three others [15–17] were prospective cohort studies. Three studies recruited only symptomatic patients postoperatively [14–16], while the two most recently published studies [17, 18] recruited all patients undergoing low anterior resection preoperatively.

Study sample sizes ranged from 8 to 137 patients. The mean age of patients was 63 years. In terms of risk factors for LARS, neoadjuvant therapy was given to 48% of patients in the four studies that reported its use, 64.4% of patients had a diverting stoma and the median timing of stoma closure ranged from 72 days to 20 months. All studies were assessed to have an overall low risk of bias. A summary of the included studies which have already been performed is shown in Table 1.

**Table 1:** Characteristics of included studies with data

Reference	Study type	Sample size (*n*)	Male:female	Mean age (years)	Neoadjuvant therapy (%)	Diverting stoma (%)	Median timing of stoma closure (range)
Van der Heijden [14]	RCT	95	59:36	63	48.4	46.3	3 months (1–5)
Harji [15]	Prospective cohort	137	88:49	65	68	92	72 days (7–538)
Darlsgaard [16]	Prospective cohort	86	52:34	63	25.6	88.4	4 months (3–7)
Olivia [17]	Prospective cohort	8	3:5	58.5	Not stated	100	20 months (13.5–35.5)
Kim [18]	RCT	42	42:0	57.6	31	52.4	Mean of 6.4 months

Given the small number of studies available, five protocols of future studies [19–23] related to education for LARS patients have been included for analysis of content and method of delivery. Three of these studies recruit all patients undergoing low anterior resection either preoperatively or prior to their ostomy reversal, while the other two recruit patients symptomatic with LARS postoperatively. This may reflect a contemporary trend towards an increasingly proactive approach to healthcare and intervention.

### Delivery and Contents of Education Intervention

The majority of education interventions were delivered in the postoperative period after sphincter-preserving surgery or ileostomy closure; only the study by Harji [15] introduced the education intervention in the preoperative period. Education was delivered in multiple methods, including in-person sessions that were either individual or in groups, phone reviews and online modules. In future study protocols, a specific information booklet for LARS and online and phone applications are being produced for RCTs [19–23]. The contents of the education interventions mainly included advice on dietary changes and toileting habits that are used to manage LARS, detailed information about LARS itself and the functional outcomes that can be expected postoperatively, as well as disease experience of other similar patients [14–18]. The length of the education intervention varied, ranging from 2 to 12 months after the initial operation. A summary of the education interventions and follow-up is shown in Table 2.

**Table 2:** Summary of education interventions

Reference	Study design	Intervention	Control	Timepoint of introduction	Length of follow-up	Education content	Outcome measures
Van der Heijden [14]	RCT	Usual care AND pelvic floor rehabilitation (PFR) - Pelvic floor muscle training - Biofeedback - Functional electrostimulation - Rectal balloon training	Usual care - Use of bulking agents - Advice on lifestyle, fluid intake, fibres, diet and toilet posture	3 months after LAR or within 6 weeks after stoma closure	3 months	Advice on lifestyle, fluid intake, fibres, diet and toilet posture	LARS score mean difference: PFR − 2.4 vs. control − 2.3, *p* = 0.93 Wexner score mean difference: PFR − 2.3 vs. control − 1.3, *p* = 0.13 Quality of Life (EORTC QLQ-CR29): no significant differences in all domains
Harji [15]	Prospective cohort study	BOREAL programme - Patient education initiative - Step 0: anti-diarrhoeal drugs + specific dietary advice - Step 1: addition of bulking agents - Step 2: PFMT, biofeedback and TAI - Step 3: SNS - Step 4: percutaneous endoscopic caecostomy + anterograde enema - Step 5: colostomy	N/A	Preoperative for total mesorectal excision	12 months: Step 0: hospital discharge Step 1: 30 days after discharge Thereafter assessments at 3, 6, 9 and 12 months	In-depth discussion about possible spectrum of postoperative functional outcomes and possible treatment options	Prevalence of minor LARS from 3 to 12 months: 14 to 6% Prevalence of major LARS from 3 to 12 months: 48 to 12% Wexner score > 4 from 3 to 12 months: 59 to 22% Quality of life (EQ-5D > 80): no LARS 57% vs. LARS 54%, *p* = 0.773
Dalsgaard [16]	Prospective cohort study	Nurse-led clinic: Assessment Dietary advice and advice on toilet routines Medications + pelvic floor training as needed TAI + / − biofeedback + / − SNS if not responding	N/A	At first visit, post primary restorative resection	Review in 2 months to offer more treatment	Dietary advice and advice on toilet routines	Prevalence of major LARS after basic treatment: 97.7% vs. 56.8% Median LARS score after basic treatment: 36 vs. 31, *p* < 0.001
Olivia [17]	Prospective cohort study	Web-based application eLARS - Provides educational content - Peer support to rectal cancer survivors with LARS	N/A	> 1 year post restorative proctectomy	2 months	Understanding LARS eModule Educational videos by specialists discussing various LARS-related topics Advice provided by peers through online forum	Patient interviews Satisfaction questionnaire App usage: 75% of participants > 4 times per month
Kim [18]	RCT	Standard care AND Bowel function improvement programme - PFMT - Exercise and diet diary - Exercise coaching - Emotional support	Standard care - Group education on postoperative care - Individual education on postoperative diet management and exercise	First session: before discharge post low anterior resection or ileostomy reversal	4-week intensive programme: - Second session—at first OPC after surgery - Third and fourth session—phone coaching sessions 3 and 4 weeks post discharge 8-week maintenance programme—text messages to increase PFMT adherence	PPT slideshow for 30 min - Function of rectum - Definition of LARS - Disease experience of rectal cancer surgery patients - Purpose and method of PFMT - Diet management - Postoperative symptom management PFMT videos	Mean LARS score after 3 months: intervention 25.67 vs. control 33.19, *p* = 0.03 Prevalence of major LARS after 3 months: intervention 52.4% vs. control 71.4% Self-efficacy scale after 3 months: intervention 64.86 vs. 63.52, *p* = 0.71 Quality of life (EORTC QLQ CR29): no significant differences in all domains except abdominal pain (intervention 1.59 vs. 9.52, *p* = 0.04)
Moon [19]	RCT protocol	Interactive online information and peer support app	Informational booklet about LARS	Post restorative proctectomy and completed all treatment in last 3 years	6 months	LARS informational modules Participants can post their LARS-related stories or questions Forum facilitated by expert healthcare professionals	PROMs Health-related QoL via EORTC QLQ-C30 Patient activation (PAM-13) Bowel function (LARS score)
Li [20]	RCT protocol	‘E-Bowel Safety’ Applet + remote weekly counselling	Information booklet that provides same information as ‘e-bowel safety’ module + standard LARS counselling at local hospital	1–3 months after sphincter-preserving surgery or ostomy closure	3-month intervention + 3-month follow-up	Symptom experience module—patient experiences and peer interaction Health diary Symptom management module Physical training information	Quality of life via EORTC QLQ-C30 Bowel function (LARS score) Self-management (BSSBQ) Social support (PSSS) Patient opinions
Sacomori [21]	RCT protocol	Pelvic floor intervention - Stage 1: prehabilitation + health education - Stage 2: rehabilitation including pelvic floor exercises and biofeedback	Standard care	Prior to sphincter-preserving surgery	Intervention until 1 month post stoma reversal, follow-up for 1 year post rehabilitation	Adequate positions for evacuation Dietary advice Bowel habit advice	Bowel symptoms (ICIQ-B and LARS score) Anorectal manometry QoL via EORTC QLQ-C30 Adherence to exercises
Powell-Chandler [22]	Prospective cohort study Protocol	Pelvic floor rehabilitation - Preoperative educational session - Qualitative interview - PFR programme	N/A	Prior to anterior resection	3 months post anterior resection	Overview of normal anatomy and bowel function How bowel function is changed by surgery Anterior resection syndrome and management	Adherence to PFR Pelvic floor tone Bowel function (LARS score) QoL via EQ-5D and EORTC QLQ-C30
Garfinkle [23]	RCT protocol	LARS patient-centred programme - Informational booklet - Patient diary - Nursing support	Standard care - Only receive Colorectal Cancer Association of Canada module on ‘Living with Colorectal Cancer’ - LARS counselling from local hospital	Post restorative proctectomy, 1 month prior to ileostomy closure	12 months after ileostomy closure	Postoperative bowel dysfunction Dietary advice Management strategies	QoL via EORTC QLQ-C30 Bowel function (LARS score) Patient activation (PAM-13) Symptom changes (MYMOP2)

### Comparison and Co-interventions

In the included RCTs, the control groups received standard care, which included the education intervention, while the intervention groups received pelvic floor muscle training (PFMT) alongside standard care [14, 18]. In two of the prospective cohort studies [15, 16], patients received stepwise care depending on the severity and response of their symptoms, starting from educational intervention only, then medications, PFMT and transanal irrigation (TAI), and finally surgical intervention. In future RCTs, online and phone applications will be compared to a standard written information booklet for LARS [19–23].

### Impact on LARS

Four of the five included studies [14–16, 18] described the impact of their interventions on LARS. Van der Heijden et al. [14] reported no significant difference in the change of LARS score in the pelvic floor rehabilitation (PFR) group compared to standard care (mean difference: PFR − 2.4 vs. control − 2.3, *p* = 0.93). Kim et al. [18] reported an average difference in LARS score of − 2.28 in the intervention group compared to the control group, and a lower prevalence of major LARS in the intervention group at 3 months (52.4% vs. 71.4%). Harji et al. [15] reported that with their rehabilitation programme, the prevalence of major LARS decreased from 48 to 12%, and the prevalence of Wexner score > 4 decreased from 59 to 22% in 12 months. Seventy-seven percent of patients from Harji’s cohort only required education and anti-diarrhoeal drugs to improve their LARS symptoms during the 12-month follow-up period. Dalsgaard et al. [16] reported that 51.2% of their patients required basic treatment, i.e. education and medications only, and within this group, the prevalence of major LARS decreased from 97 to 57%.

### Quality of Life

Quality of life measures were reported by three studies [14, 15, 18]. The two RCTs used the European Organisation of Research and Treatment of Cancer (EORTC QLQ-CR29) questionnaire to assess quality of life, while Harji et al. used the EQ-5D questionnaire. Van der Heijden et al. [14] reported no significant difference between the two groups in the final EORTC scores, but there was a clinical difference in favour of the intervention group in relevant domains such as anxiety and urinary frequency. Kim et al. [17] also reported no significant differences between the two groups in all functional and symptom scores of the EORTC questionnaire, except for lower abdominal pain scores in the intervention group. Harji et al.’s study [15] only measured quality of life at the end of follow-up and found no difference in EQ-5D questionnaire scores between patients with major or minor LARS.

Olivia et al.’s study [17] reported descriptive outcomes after implementing their online educational modules for LARS. Their participants described that the online educational module provided credible information on LARS, which was delivered at an appropriate reading level and had useful accompanying illustrations. The patient forum on the online module allowed participants to share their own experiences and provide emotional support to one another. A satisfaction survey was provided at the end of their follow-up, and seven of the eight participants were ‘mostly’ or ‘very’ satisfied with the information provided by the online educational modules, and all eight participants would recommend it to other rectal cancer survivors with LARS.

## Discussion

There is increasing awareness of LARS and its impact not only on functional outcomes but also on a patient’s quality of life [2]. LARS is associated with functional symptoms such as diarrhoea and incontinence, which in turn can lead to social withdrawal, sleep pattern disturbances and mental distress, ultimately leading to poorer quality of life [24]. Previous studies with patient interviews suggest that there is minimal preoperative or postoperative education about LARS, and counselling provided by clinicians on LARS largely surrounded pharmacological management rather than education about the expected functional change or psychosocial impact of LARS [11, 25]. Given the lack of credible resources and treatment protocols for LARS, rectal cancer patients require more access to better information and counselling about LARS in order to manage their symptoms more effectively [9–11].

More recent studies recognise that the treatment of LARS is most effective when detected and initiated early, leading to the development of the MANUEL and BOREAL protocols, and this is another area where perioperative education can really come to the fore, prompting patients to seek attention and treatment early, and encouraging clinicians to systematically step up treatment modalities as required [7, 15].

Education interventions for colorectal cancer patients with stomas have been shown to provide improvements in psychosocial adjustment and self-management skills with a stoma. Overall costs after patient education interventions were also less than standard care [12]. To our knowledge, this is the first systematic review of education interventions in colorectal cancer patients with LARS.

Our results suggest that education interventions have a role to play in improving the symptoms of LARS, or at least in mitigating its impact on patients’ lives. In the included RCTs, even the control groups that only received an education intervention experienced improvements in the mean LARS scores. Most patients within the Harji and Dalsgaard cohort studies experienced significant improvement in LARS symptoms with only perioperative education and use of medical therapy, indicating both the significance of early and appropriate therapy, which is an outcome that is hoped to be achieved through education. In terms of quality of life, there were no significant differences between the intervention and control groups for the EORTC QLQ-CR29 questionnaires, suggesting that education alone can be beneficial in improving the quality of life in a patient with LARS.

These marginal gains must not be overlooked, as the implementation of an educational intervention is cost-effective and easily disseminated, making it a crucial component for LARS intervention in any setting, regardless of resource constraints.

As alluded to previously, the multi-modality nature of the treatment of LARS is such that although each intervention can contribute to symptom improvement incrementally when used to its fullest, the reality of practicing within a resource-limited healthcare system is that not every centre (and thus not every patient) will have access to resources to implement more resource intensive treatments for LARS such as PFMT, TAI or SNS [26]. Therefore, it is important to study each component both individually and in tandem to identify their impact on LARS. Future studies looking at education interventions alone for LARS will ideally provide more information on its impact. However, we also acknowledge that this may be impractical, as many patients may require escalation in therapy in order to improve their LARS symptoms.

The different modes of education delivery and dissemination seen in the included studies also show that education for LARS is becoming more accessible to patients. In the post COVID-19 pandemic era, many domains of medicine have developed online and remote methods of disseminating education [27]. Through the use of mobile applications, online modules and remote reviews with clinicians, patients experiencing LARS will be able to receive the education and counselling even if they are socially isolated [17–20, 23]. An additional benefit of online resources is that patients with LARS can become connected and support one another through their experiences [17].

Overall, there is a paucity of research in the use of education interventions in LARS, despite the potential benefits that they can provide. A significant limitation to this review is the lack of comparison of education interventions to a control group, given that through the natural progression of LARS, it is anticipated that there is some symptom improvement over time; however, its magnitude is yet unclear. Further research should ideally assess standardised outcomes, such as changes in LARS scores, effects of patient-reported outcome measures and quality of life measures (e.g. EORTC questionnaires), in order for data to be meta-analysed in the future.

## Conclusion

This systematic review demonstrates that education interventions have a role to play in reducing the prevalence of LARS following resection for rectal cancer. This can be delivered in many ways but generally includes education about the pathophysiology of the disease, dietary and lifestyle modifications to improve bowel function, as well as potential interventions that can be undertaken in a step up fashion. It is a cost-effective, easily initiated intervention, which is often studied in conjunction with other co-interventions. However, the marginal gains that can be derived from it must not be overlooked, and given the paucity of studies focused on the topic, there is significant potential for further research to determine its true effect, as well as optimal content and delivery methods.

## Electronic supplementary material

Below is the link to the electronic supplementary material.

Supplementary file1 (DOCX 13 KB)

Supplementary file2 (DOCX 13 KB)

## Data Availability

The datasets generated during and/or analysed during the current study are available from the corresponding author on reasonable request.

## Appendix

Example of PubMed search strategy.

1. Low anterior resection syndrome (MeSH terms)
2. Awareness
3. Management
4. Education
5. Strategy*
6. Tool*
7. #2 OR #3 OR #4 OR #5 OR #6
8. Patient
9. Clinician*
10. Surgeon*
11. #8 OR #9 OR #10
12. #1 AND #7 AND #11

### Searched Databases

EMBASE, MEDLINE, Web of Science and Google Scholar, CINAHL Cochrane, mRCTs or International Clinical Trials Registry Platform (ICTRP). Here, we propose 12 databases (PubMed, Scopus, Web of Science, EMBASE, GHL, VHL, Cochrane, Google Scholar, Clinical trials.gov, mRCTs, POPLINE and SIGLE).

### Inclusion Criteria

1. Any article evaluating clinician awareness or management of LARS.
2. Any article evaluating patient awareness and education resources for LARS.
3. No restriction regarding country, clinician type, patient age, race, gender, publication language and date.

### Exclusion Criteria

1. Abstract only papers as preceding papers, conference, editorial and author response theses and books.
2. Articles without full-text availability.
3. Studies with data not reliably extracted, duplicate or overlapping data.

## References

[1] Sun R, Dai Z, Zhang Y et al (2021) The incidence and risk factors of low anterior resection syndrome (LARS) after sphincter-preserving surgery of rectal cancer: a systematic review and meta-analysis. Support Care Cancer 29(120):7249–7258 34296335 10.1007/s00520-021-06326-2

[2] Keane C, Fearnhead N, Bordeianou L et al (2020) International consensus definition of low anterior resection syndrome. Anz J Surg 90(1):300–307 32040983 10.1111/ans.15421

[3] Emmertsen K, Laurberg S (2013) Impact of bowel dysfunction on quality of life after sphincter-preserving resection for rectal cancer. Br J Surg 100(10):1377–1387 23939851 10.1002/bjs.9223

[4] Emmertsen K, Laurberg S (2012) Low anterior resection syndrome score: development and validation of a symptom-based scoring system for bowel dysfunction after low anterior resection for rectal cancer. Ann Surg 255(5):922–928 22504191 10.1097/SLA.0b013e31824f1c21

[5] Pieniowski E, Nordenvall C, Palmer G et al (2020) Prevalence of low anterior resection syndrome and impact on quality of life after rectal cancer surgery: population-based study. BJS Open 4(5):935–942 32530135 10.1002/bjs5.50312 PMC7528525

[6] Messick C, Boutros M (2021) Low anterior resection syndrome: future directions in treatment and prevention. Semin Colon Rectal Surg 32(4):100850

[7] Christensen P, Baeten C, Espin-Basany E et al (2021) Management guidelines for low anterior resection syndrome – the MANUEL project. Colorectal Dis 23(2):461–475 33411977 10.1111/codi.15517 PMC7986060

[8] Brock H, Lambrineas L, Ong H et al (2023) Preventative strategies for low anterior resection syndrome. Tech Coloproctol 28(1):10. 10.1007/s10151-023-02872-5 38091118 10.1007/s10151-023-02872-5

[9] Garfinkle R, Wong-Chong N, Petrucci A et al (2019) Assessing the readability, quality and accuracy of online health information for patients with low anterior resection syndrome following surgery for rectal cancer. Colorectal Dis 21(5):523–531 30609222 10.1111/codi.14548

[10] Pape E, Decoene E, Debrauwere M et al (2023) Information and counselling needs of patients with major low anterior resection syndrome: a qualitative study. J Clin Nurs 32(7–8):1240–1250 35253296 10.1111/jocn.16277

[11] Burch J, Wright J, Taylor C et al (2023) ‘He’s a surgeon, like I’m not going to waste his time’: interviews to determine healthcare needs of people with low anterior resection syndrome after rectal cancer surgery. Colorectal Dis 25(5):880–887 36633117 10.1111/codi.16475

[12] Faury S, Koleck M, Foucaud J et al (2017) Patient education interventions for colorectal cancer patients with stoma: a systematic review. Patient Educ Couns 100(10):1807–1819 28602564 10.1016/j.pec.2017.05.034

[13] Danielsen A, Burcharth J, Rosenberg J (2013) Patient education has a positive effect in patients with a stoma: a systematic review. Colorectal Dis 15(6):e276–e283 23470040 10.1111/codi.12197

[14] Van der Heijden J, Kalkdijk-Dijkstra A, Pierie J et al (2022) Pelvic floor rehabilitation after rectal cancer surgery. Ann Surg 276(1):38–45 34966064 10.1097/SLA.0000000000005353

[15] Harji D, Fernandez B, Boissieras L et al (2021) A novel bowel rehabilitation programme after total mesorectal excision for rectal cancer: the BOREAL pilot study. Colorectal Dis 23:2619–2626 34264005 10.1111/codi.15812

[16] Daarlsgard P, Emmertsen K, Mekhael M et al (2020) Nurse-led standardised intervention for low anterior resection syndrome: a population-based pilot study. Colorectal Dis 23(2):434–443 10.1111/codi.15497 33340218

[17] Olivia M, Allister S, Moon J et al (2023) An online educational and supportive care application for rectal cancer survivors with low anterior resection syndrome: a mixed methods pilot study. Colorectal Dis 25:1812–1820 37501348 10.1111/codi.16665

[18] Kim Y, Oh E, Chu S et al (2023) Effects of a bowel function improvement program for patients with rectal cancer surgery: a randomised controlled trial. Eur J Oncol Nurs 66:102382 37542970 10.1016/j.ejon.2023.102382

[19] Moon J, Monton O, Smith A et al (2021) Interactive online information and peer support application for patients with low anterior resection syndrome: patient survey and protocol for a multicentre randomised controlled trial. Colorectal Dis 23:1248–1257 33638278 10.1111/codi.15602

[20] Li H, Zhou P, Pang X et al (2022) Mobile health-based remote interaction management intervention for patients with low anterior resection syndrome: study protocol for a randomised controlled trial. BMJ Open 12(12):e066046 36564113 10.1136/bmjopen-2022-066046 PMC9791376

[21] Sacomori C, Lorca L, Martinez-Mardones M et al (2021) A randomized clinical trial to assess the effectiveness of pre- and post-surgical pelvic floor physiotherapy for bowel symptoms, pelvic floor function, and quality of life of patients with rectal cancer: CARRET protocol. Trials 22(1):448 34256795 10.1186/s13063-021-05396-1 PMC8276537

[22] Powell-Chandler A, Rees B, Broad C et al (2018) Physiotherapy and anterior resection syndrome (Paris) trial: feasibility study protocol. BMJ Open 8(6):e021855 29961031 10.1136/bmjopen-2018-021855 PMC6042554

[23] Garfinkle R, Loiselle C, Park J et al (2020) Development and evaluation of a patient-centred program for low anterior resection syndrome: protocol for a randomized controlled trial. BMJ Open 10(5):e035587 32474427 10.1136/bmjopen-2019-035587 PMC7264642

[24] Pape E, Pattyn P, Van Hecke A et al (2021) Impact of low anterior resection syndrome on the quality of life and treatment options of LARS – a cross sectional study. Eur J Oncol Nurs 50:101878 33246248 10.1016/j.ejon.2020.101878

[25] Taylor C, Bradshaw E (2013) Lived experiences of altered bowel function (anterior resection syndrome) after temporary stoma reversal. J Wound Ostomy Continence Nurs 40(4):415–421 23820474 10.1097/WON.0b013e318296b5a4

[26] Lambrineas L, Brock H, Ong H et al (2024) Challenges in evaluating pelvic floor physiotherapy based strategies in low anterior resection syndrome: a systematic review and qualitative analysis. Colorectal Dis 26(2):258–271. 10.1111/codi.16839 38173138 10.1111/codi.16839

[27] Keller D, Grossman R, Winter D (2020) Choosing the new normal for surgical education using alternative platforms. Surgery (Oxf) 38(10):617–622 32904575 10.1016/j.mpsur.2020.07.017 PMC7456587

