# Supplementary material for: The Role of Patient Education in Low Anterior Resection Syndrome: A Systematic Review
Source: J Cancer Educ. 2025 Feb 27;40(5):650–9. doi: 10.1007/s13187-025-02593-3 (PMC12504328; doi:10.1007/s13187-025-02593-3)
Supplement: Supplementary file 1 — Supplementary file1 (DOCX 13 KB) [file 13187_2025_2593_MOESM1_ESM.docx]

| Study | Confounding | Selection | Intervention Classification | Deviation from intervention | Missing data | Measurement of outcome | Selection of reported result | Overall |
| --- | --- | --- | --- | --- | --- | --- | --- | --- |
| Olivia^17^ 2023 | Low | Low | Low | Low | Low | Low | Low | Low |
| Harji^15^ 2021 | Low | Low | Low | Moderate | Low | Low | Low | Low |
| Dalsgaard^16^ 2021 | Low | Low | Low | Low | Moderate | Low | Low | Low |

Supplementary Table 1: Assessment of Risk of Bias for Non-Comparative Studies using ROBINS-I Tool
